# Metformin Activates the Protective Effects of the AMPK Pathway in Acute Lung Injury Caused by Paraquat Poisoning

**DOI:** 10.1155/2019/1709718

**Published:** 2019-10-30

**Authors:** Liaozhang Wu, Yifang Cen, Menglong Feng, Yanna Zhou, Hui Tang, Xueli Liao, Yanze Wang, Min Wang, Manhong Zhou

**Affiliations:** ^1^Department of Emergency, The Affiliated Hospital of Zunyi Medical University, Zunyi 563003, China; ^2^Department of Rehabilitation, The Affiliated Hospital of Zunyi Medical University, Zunyi 563003, China; ^3^Department of Epidemiology and Health Statistics, School of Public Health, Zunyi Medical University, Zunyi 563006, China

## Abstract

**Objective:**

To observe whether metformin (MET) plays a protective role in acute lung injury (ALI) induced by paraquat (PQ) poisoning in rats by activating the AMPK/NF-*κ*B signaling pathway.

**Methods:**

PQ exposure was used to construct a rat model of ALI and a model of acute type II alveolar epithelial cell (RLE-6TN) injury, and MET intervention was performed. Rat lung tissue samples were collected to evaluate pathological changes in rat lung tissue, the oxidation index, and inflammatory factors; cell viability was detected by CCK-8 assays, and the protein expression levels of phospho-AMPK and phospho-NF-*κ*Bp65 in rat lung tissue and RLE-6TN cells were observed by Western blotting.

**Results:**

Compared with the PQ group, the MET treatment group showed significantly (1) reduced lung wet/dry ratio (W/D: 4.67 ± 0.31 vs. 5.45 ± 0.40, *P* < 0.001), (2) reduced pathological changes in lung tissue, (3) decreased MDA levels (nmol/mg prot: 2.70 ± 0.19 vs. 3.08 ± 0.15, *P* < 0.001) and increased SOD and GSH-Px activities (U/mg prot: 76.17 ± 5.22 vs. 45.23 ± 6.58, 30.40 ± 2.84 vs. 21.00 ± 3.20; all *P* < 0.001) in lung tissue homogenate, (4) reduced levels of IL-1*β*, IL-6, and TNF-*α* in lung tissue homogenates (pg/mL: 47.87 ± 5.06 vs. 66.77 ± 6.55; 93.03 ± 7.41 vs. 107.39 ± 9.81; 75.73 ± 6.08 vs. 89.12 ± 8.94; all *P* < 0.001), (5) increased activity of RLE-6TN cells (%: 0.69 ± 0.09, 0.76 ± 0.06, and 0.58 ± 0.03 vs. 0.50 ± 0.05; all *P* < 0.05), (6) decreased protein levels of phospho-NF-*κ*Bp65 in lung homogenates and RLE-6TN cells (p-NF-*κ*B/NF-*κ*B: 0.47 ± 0.09 vs. 0.81 ± 0.13; 0.26 ± 0.07 vs. 0.79 ± 0.13; all *P* < 0.01), and (7) upregulated protein expression of phospho-AMPK in lung homogenates and RLE-6TN cells (p-AMPK/AMPK: 0.88 ± 0.05 vs. 0.36 ± 0.12; 0.93 ± 0.03 vs. 0.56 ± 0.15; all *P* < 0.01). After the addition of the AMPK inhibitor Compound C (Com C), the protein expression levels of phospho-AMPK and phospho-NF-*κ*Bp65 returned to baseline.

**Conclusion:**

MET can effectively alleviate ALI induced by paraquat poisoning and increase the viability of cells exposed to paraquat. The mechanism may be related to the activation of the AMPK/NF-*κ*B pathway, downregulation of inflammatory mediators such as IL-6 and TNF-*α*, and upregulation of the SOD and GSH-Px oxidation index, and these effects can be inhibited by the AMPK inhibitor Com C.

## 1. Introduction

Paraquat (PQ, 1,1-dimethyl-4,4-bipyridinium dichloride) belongs to the class of bipyridine herbicides. Because of its characteristics such as broad spectrum activity, high efficiency, limited environmental pollution, and ease of use [1], it is widely used in agricultural production worldwide, especially in developing countries. However, acute poisoning caused by accidental or intentional ingestion often occurs. Although PQ is highly toxic to humans and livestock, there is currently no specific antidote. The mortality rate after poisoning is as high as 60%-70% [2]. Therefore, PQ poisoning is still a major problem in the field of emergency medical research, and it is an important public health problem.

PQ accumulates in the lungs, which leads mainly to lung injury. The main manifestations of PQ exposure include acute lung injury (ALI) and pulmonary fibrosis [3], which are the main causes of death by PQ poisoning. It has been previously reported [4, 5] that oxidative stress and inflammatory reactions are involved in the early stage of PQ-induced ALI. AMP-activated protein kinase (AMPK) is a highly expressed and highly conserved serine/threonine protein kinase in eukaryotes that senses energy changes in the body and plays an important role in energy regulation [6]. In addition to participating in energy metabolism, AMPK activation is also involved in combating oxidative stress [7] and reducing inflammation [8]. Many in vitro experiments have also demonstrated that lipopolysaccharide-induced inflammatory responses can be inhibited by activating AMPK with AICAR [9]. Although the activation of AMPK has been shown to have anti-inflammatory effects, there is little information concerning the role of AMPK in modulating neutrophil function and neutrophil-dependent inflammatory events, such as ALI.

Metformin (MET) is the first-line treatment for type 2 diabetes [10]. Piwkowska et al. reported that MET can reduce intracellular energy and thus activate the AMPK signaling pathway by inhibiting complex I of the electron transport chain [11]. It has also been reported that MET can inhibit mTOR by activating the AMPK signaling pathway and can exert anti-inflammatory effects in endotoxin-induced ALI [12]. Zhang et al. [13] found that in lipopolysaccharide- (LPS-) induced ALI, MET reduced lung tissue damage by promoting the expression of AMPK*α*1 in the lung tissue and reducing the release of inflammatory cytokines. Therefore, MET has a role in alleviating oxidative damage and exerting anti-inflammatory effects. The mechanism may be related to the activation of AMPK, which has been confirmed in different types of ALI; however, it remains unknown whether MET is protecting in PQ-induced ALI. At present, there are no relevant literature reports. This study is aimed at determining whether MET can protect against PQ-induced ALI in a rat model and preliminarily exploring the mechanism through in vivo and in vitro experiments to provide a theoretical basis and potential therapeutic targets for the clinical treatment of PQ poisoning-induced ALI.

## 2. Materials and Methods

### 2.1. Experimental Animals and Materials

#### 2.1.1. Animals and Cell Lines

Forty adult male SD rats, weighing 200 ± 20 g, were purchased from Changsha Tianqin Biotechnology Company (experimental animal license number SCXK (Xiang) 2014-0011). Rats were housed at the Experimental Animal Center of Zunyi Medical University (20-25°C, 50 ± 5% relative humidity, 12 hour/12 hour day/night cycle), fed standard chow and water, and acclimated for at least one week before starting experiments. The animal experiments in this study were approved by the Animal Experimental Ethics Committee of Zunyi Medical College (2014-2-075).

Rat type II alveolar epithelial cells (RLE-6TN) were purchased from Shanghai Sixin Biotechnology Company (Shanghai, China). RLE-6TN cells were plated at a density of 4 × 10^4^ cells/mL and cultured in an incubator (37°C, 5% CO_2_) with RPMI 1640 medium containing 10% fetal bovine serum; the medium was changed every 2 days, and the cells were passaged 1 : 3 upon reaching 80% confluence.

#### 2.1.2. Antibodies and Reagents

The following reagents were used in this study: dichloro-paraquat, metformin hydrochloride, and Com C (Sigma-Aldrich, USA); HE staining reagent (Department of Pathology, Affiliated Hospital of Zunyi Medical University, China); murine IL-1*β* (No. E-EL-H0149c), IL-6 (No. E-EL-H0102c), and TNF-*α* (No. E-EL-H0109c) ELISA kits (Wuhan Sanying Biotechnology Co., Ltd., China); SOD (No. A001-3), GSH-Px (No. A005-1-1), and MDA (No. A003-2) kits (Nanjing Institute of Bioengineering, China); fetal bovine serum (Gemini, USA); 0.25% trypsin-EDTA and RPMI 1640 culture medium (HyClone, USA); streptomycin solution, CCK-8 assays, and BCA kit (Beijing Soleil Technology Co., Ltd., China); cell lysis solution (Shanghai Biyuntian Biotechnology Research Institute, China); rabbit anti-mouse AMPK-*α*1 antibody, rabbit anti-mouse p-AMPK-*α*1 antibody, rabbit anti-mouse NF-*κ*B p65 antibody, rabbit anti-mouse NF-*κ*Bp65 (pS536) antibody, mouse anti-*β*-actin antibody, HRP-labeled goat anti-rabbit IgG antibody, and HRP-labeled goat anti-mouse IgG antibody (Beijing Boaosen Biotechnology Co., Ltd., China); chemiluminescence fluorescence imaging system (Bio-Rad, USA); and fully automated multifunction microplate reader (Thermo Fisher Scientific, USA).

### 2.2. Experimental Design

#### 2.2.1. Establishment of an Animal Model of Metformin Intervention

SD rats were randomly divided into the following 4 groups according to the random number table method. (1) Blank control group (control group, *n* = 10): 1 mL of normal saline was injected into the left peritoneal cavity, and 2 mL of normal saline was administered after 2 hours. (2) Paraquat poisoning group (PQ group, *n* = 10): paraquat was diluted to 10 mg/mL in normal saline, and animals were treated with 30 mg/kg PQ diluted to 1 mL with physiological saline. Two hours after intraperitoneal injection, 2 mL of normal saline was administered intragastrically. (3) Metformin control group (MET group, *n* = 10): 1 mL of normal saline was injected into the peritoneal cavity on the left side, and metformin hydrochloride was diluted with physiological saline to 50 mg/mL; this solution at 400 mg/kg was diluted to 2 mL with physiological saline and administered intragastrically 2 hours later. (4) Metformin treatment group (PQ+MET group, *n* = 10): 2 hours after the peritoneal injection (left side) of 1 mL of PQ solution, 2 mL of MET solution was administered intragastrically. All rats received an intragastric treatment once a day for 7 days at a fixed time every morning. After 7 days, the rats were sacrificed, and lung tissue samples were harvested.

#### 2.2.2. Establishment of Cell Model of Metformin Intervention

RLE-6TN cells were divided into the following 5 groups: (1) blank control group (control group): cells in 0.8 mL of medium were treated with 1.2 mL of PBS; (2) paraquat injury group (PQ group): cells in 0.8 mL of medium were treated with 0.8 mL of PBS and 0.4 mL of PQ (final concentration, 500 *μ*mol/L); (3) metformin group (MET group): cells in 0.8 mL of medium were treated with 0.8 mL of PBS and 0.4 mL MET (final concentration, 2.5 mmol/L); (4) metformin intervention group (PQ+MET group): cells in 0.8 mL of medium were pretreated for 2 hours with 0.4 mL of PBS and 0.4 mL MET and then treated with 0.4 mL of PQ; (5) Compound C group (PQ+MET+Com C group): cells in 0.8 mL of medium were pretreated with 0.4 mL of Com C (final concentration, 250 *μ*mol/L) and 0.4 mL MET for 2 hours and then treated with 0.4 mL of PQ. After all groups of cells were incubated for 24 hours in the incubator, the cells were harvested and protein was obtained.

### 2.3. Lung Tissue Transmission Electron Microscopy

Immediately after anesthesia, the chest of the rats was opened, the lungs were fully exposed, and a small piece of lung tissue was quickly removed from the left lower lobe. The lung tissue samples were fixed in 2.5% glutaraldehyde, cut to 1 mm^3^, fixed at 4°C for more than 4 hours, placed in 1% osmic acid, fixed for 1.5 hours, and dehydrated with different concentrations of ethanol and acetone. Then, the tissues were soaked, embedded, trimmed, sectioned, stained, and observed by TEM.

### 2.4. Determination of Lung W/D

After the rats were sacrificed, the right upper lobe was washed 3 times with sterile saline. The filter paper was blotted and weighed. After the tissues were dried at 80°C for 48 h, the dry weight was determined, and the lung wet/dry weight ratio was calculated to reflect the severity of lung edema.

### 2.5. Lung Histopathology

Lung tissue was taken from the right lower lobe of the rat lung and fixed with 4% paraformaldehyde for 72 hours. After paraffin embedding, sectioning, and dewaxing, the sections were stained with hematoxylin and eosin. The pathological changes in the lung tissues were observed under a light microscope. Lung injury scores were calculated according to the method described by Mikawa et al. [14]. The severity of lung injury was graded 0-4 on the following four aspects: (1) alveolar congestion; (2) hemorrhage; (3) infiltration of neutrophils in the alveolar space or blood vessel wall; and (4) thickening of the alveolar wall and/or formation of a transparent film. The lung injury score was calculated as the sum of the score of each indicator.

### 2.6. Determination of Oxidative Index and Inflammatory Factors in Rat Lung Tissue Homogenates

Each sample was processed according to the instructions of the ELISA kit, and the contents of IL-1*β*, IL-6, and TNF-*α* were detected separately. In the experiments, blank control wells and standard wells were set, and the absorbance at 450 nm was determined. The standard absorbance value and corresponding concentration value were used to make a standard curve. The concentration value of the test sample was calculated from the standard curve. The activity of SOD and GSH-Px and the content of MDA were determined by the thiobarbituric acid, enzymatic reaction glutathione depletion, and xanthine oxidase methods according to the SOD, GSH-Px, and MDA kit instructions.

### 2.7. RLE-6TN Cell Survival (CCK8 Assay)

RLE-6TN cells in the logarithmic growth phase were uniformly seeded in 96-well plates (100 *μ*L/well). The 96-well plates were kept in the incubator until the cells attached to the wells. The cells were pretreated with different concentrations of MET (1.25, 2.5, 5, and 10 mmol/L) for 2 hours and then incubated with PQ for 24 hours. Ten microliters of CCK-8 solution was added to each well, the 96-well plates were further incubated in the incubator for 2 hours, and the absorbance at 450 nm was measured.

### 2.8. Western Blotting Analysis of Rat Lung Tissue and RLE-6TN Cells

Rat lung tissue and RLE-6TN cells were lysed with appropriate amounts of lysis buffer and centrifuged at 12000 r/min at 4°C for 10 min, and the supernatant was obtained. The protein concentration was determined by the BCA method, and samples were diluted1/4 (*v*/*v*) with loading buffer and incubated at 100°C for 5 min to denature protein. Samples containing 20 *μ*g of total protein were added to each lane for SDS-PAGE. After electrophoresis, the proteins were transferred to PVDF membranes at 300 mA for 70 min, and the membranes were blocked with 5% skim milk powder or 3% BSA in TBST blocking solution for 2 hours at room temperature. Then, the antibody was diluted with the corresponding primary anti-blocking solution and incubated at 4°C overnight with the membranes, which were then washed 3 times with TBST for 15 min each. The membranes were incubated with the secondary antibody for 2 hours at room temperature with shaking and then washed 3 times with TBST for 15 min each. The luminescence liquid A and B mixture was evenly dropped onto the membrane, which was placed in a Bio-Rad gel imaging system for imaging. The Western blotting results were analyzed by grayscale scanning using ImageJ software.

### 2.9. Statistical Analysis

SPSS18.0 software was used for statistical analysis. Statistical description of measurement data was expressed as the mean ± standard deviation ($\bar{x}\pm s$), and multiple sets were analyzed by one-way ANOVA. For pairwise comparison between groups, if the variance was homogeneous, the least significant difference (LSD) was used for analysis, and if the variance was not homogeneous, the Tamhane test was used for analysis. The statistical significance was set at *P* < 0.05.

## 3. Results

### 3.1. Lung Tissue Wet/Dry Weight Ratio

**Figure 1:**
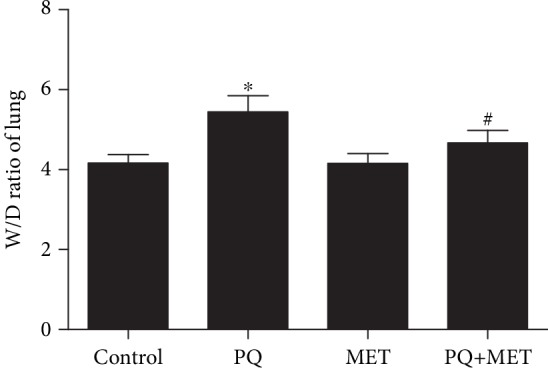
Effect of MET on the W/D ratio of lung tissue in rats treated with PQ. The PQ group received a single intraperitoneal injection of 30 mg/kg paraquat, while the control group received an intraperitoneal injection of the same volume of normal saline, and the MET and PQ+MET groups received intragastric treatment with 400 mg/kg MET (once per day for 7 days) 2 hours after model establishment. After one night of fasting, the lungs of the rats were removed, and the W/D ratio was calculated. Data are shown as the mean ± SD (*n* = 10 except for the PQ group (*n* = 8) and the PQ+MET group (*n* = 9)); *F* = 36.559, *P* < 0.001; ^∗^*P* < 0.05 vs. control group, ^#^*P* < 0.05 vs. PQ group.

To investigate the effect of metformin on pulmonary edema, we examined the lung wet/dry weight ratio in experimental rats (Figure 1). The results showed that the lung wet/dry ratio was significantly increased in the PQ group compared with the control group (*P* < 0.001) and was lower in the PQ+MET group than in the PQ group (*P* < 0.001).

### 3.2. Effects of MET on Lung Tissue Structural Changes Induced by PQ in Rats with ALI as Determined by Transmission Electron Microscopy

**Figure 2:**
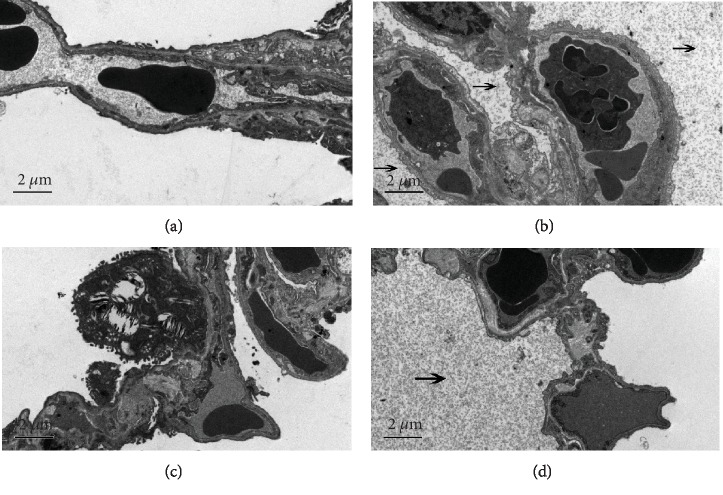
Effect of MET on changes in lung tissue structure induced by PQ in rats with ALI as determined by transmission electron microscopy. Changes of lung tissues after PQ exposure for 7 days were observed by transmission electron microscopy (magnification by 10000 times). The arrow indicates an inflammatory exudation. (a) Control group. (b) PQ group. (c) MET group. (d) PQ+MET group.

As shown in Figure 2, the lung tissue of the control and MET groups showed clear, well-ordered structures of epithelial cells, basement membrane, and endothelial cells under transmission electron microscopy; no expansion of interstitial blood vessels; a thin alveolar wall; and a clear alveolar cavity. The PQ group showed a large amount of inflammatory exudate within the alveolar cavity and swollen alveolar epithelial cells, indicating edema. The PQ+MET group showed well-defined alveolar structures, type 2 lung epithelial cells, and lamellar bodies, and some of the alveolar cavity exudate had decreased or even disappeared.

### 3.3. Lung Histopathological Observation

**Figure 3:**
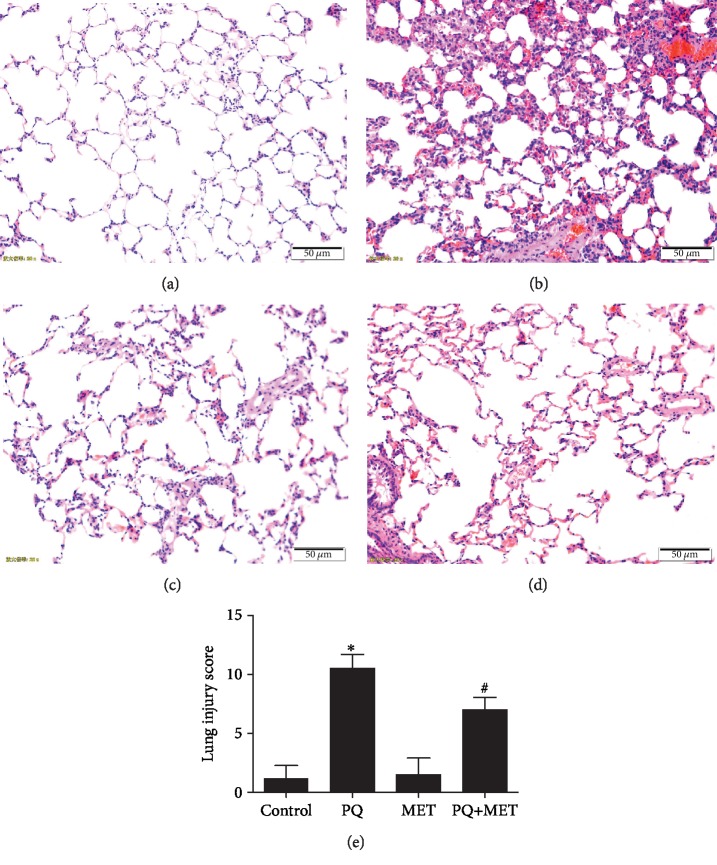
Effect of MET on lung histopathology in rats treated with PQ. The lung histological changes were determined by HE staining (a–d) and lung injury score (e) at 7 days post PQ exposure (magnification 200 times). (a) Control group. (b) PQ group. (c) MET group. (d) PQ+MET group. (e) Lung injury score. Data are shown as the mean ± SD; *F* = 114.451, *P* < 0.001; ^∗^*P* < 0.05 vs. control group. ^#^*P* < 0.05 vs. PQ group.

The lungs of the control and MET groups had well-defined structures, no expansion of interstitial blood vessels, no bleeding, no inflammatory infiltration, no thickening of the alveolar wall, a clear alveolar space, and a complete bronchial epithelium (Figures 3(a) and 3(c)). The PQ group showed extensive inflammatory cell infiltration in the pulmonary interstitial and alveolar spaces, with diffuse pulmonary hemorrhage, alveolar collapse, pulmonary interstitial edema, and extensive alveolar septal thickening (Figure 3(b)). The PQ+MET group showed a small amount of inflammatory cell infiltration within interstitial and alveolar spaces, accompanied by pulmonary hemorrhage, slight collapse of alveoli, mild alveolar sepsis, and partial alveolar septal thickening (Figure 3(d)).

### 3.4. Detection of the Oxidation Index and Inflammatory Factors in Lung Tissue Homogenate

**Figure 4:**
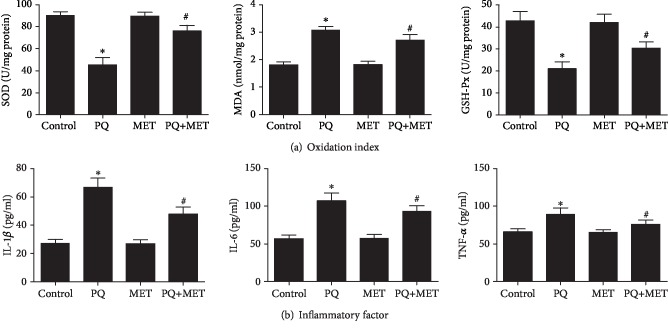
Effect of MET on the oxidation index and inflammatory factors in lung tissue homogenates from rats treated with PQ. The PQ group received a single intraperitoneal injection of 30 mg/kg paraquat, while the control group received an intraperitoneal injection of the same dose of normal saline, and the MET and PQ+MET groups received intragastric treatment with 400 mg/kg MET (once per day for 7 days) 2 hours after model establishment. The lung tissue of the rats was homogenized at a ratio of 9/1 (normal saline/lung tissue) and centrifuged at 3000 rpm and 4°C for 20 min, and the supernatant was analyzed by a relevant ELISA and a chemical kit. Data are shown as the mean ± SD; SOD: *F* = 167.637, *P* < 0.001; GSH-Px: *F* = 76.096, *P* < 0.001; MDA: *F* = 177.474, *P* < 0.001; IL-1*β*: *F* = 163.495, *P* < 0.001; IL-6: *F* = 125.042, *P* < 0.001; TNF-*α*: *F* = 31.974, *P* < 0.001; ^∗^*P* < 0.05 vs. control group, ^#^*P* < 0.05 vs. PQ group.

Compared with the control group, the PQ group showed a significant increase in MDA levels in lung tissue homogenates (*P* < 0.001), while SOD and GSH-Px activities were significantly decreased (all *P* < 0.001). After MET treatment, MDA levels were decreased in comparison with PQ alone, and SOD and GSH-Px activities were significantly higher than those with PQ alone (all *P* < 0.001) (Figure 4(a)). Compared with the control group, the levels of IL-1*β*, IL-6, and TNF-*α* in lung homogenates were significantly increased in the PQ group compared with the control group (all *P* < 0.001) but were lower in the PQ+MET group than in the PQ group (all *P* < 0.001) (Figure 4(b)).

### 3.5. Effect of MET on PQ-Induced Changes in Protein Levels in Rats with Acute Lung Injury

**Figure 5:**
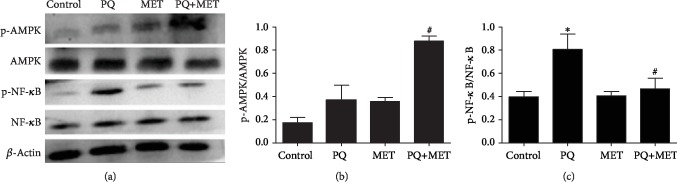
Effect of MET on the expression of phospho-AMPK and phospho-NF-*κ*Bp65 protein in the lung tissue of rats treated with PQ. The PQ group received a single intraperitoneal injection of 30 mg/kg paraquat, while the control group received an intraperitoneal injection of the same volume of normal saline, and the MET and PQ+MET groups received intragastric treatment with 400 mg/kg MET (once per day for 7 days) 2 hours after model establishment. Lysis buffer was added to rat lung tissue samples for protein extraction and Western blotting analysis. Data are shown as the mean ± SD; (b) *F* = 59.196, *P* < 0.001; (c) *F* = 15.684, *P* < 0.01; ^∗^*P* < 0.05 vs. control group, ^#^*P* < 0.05 vs. PQ group.

As shown in Figures 5(a)–5(c), the expression of phospho-AMPK protein in the lung tissue was significantly higher in the PQ+MET group than in the PQ group (*P* < 0.001). The expression of phospho-NF-*κ*Bp65 protein in the lung tissue was significantly higher in the PQ group than in the control group (*P* < 0.001) and was lower in the PQ+MET group than in the PQ group (*P* < 0.01).

### 3.6. Effect of MET on the Proliferation Rate of RLE-6TN Cells after PQ Treatment

**Figure 6:**
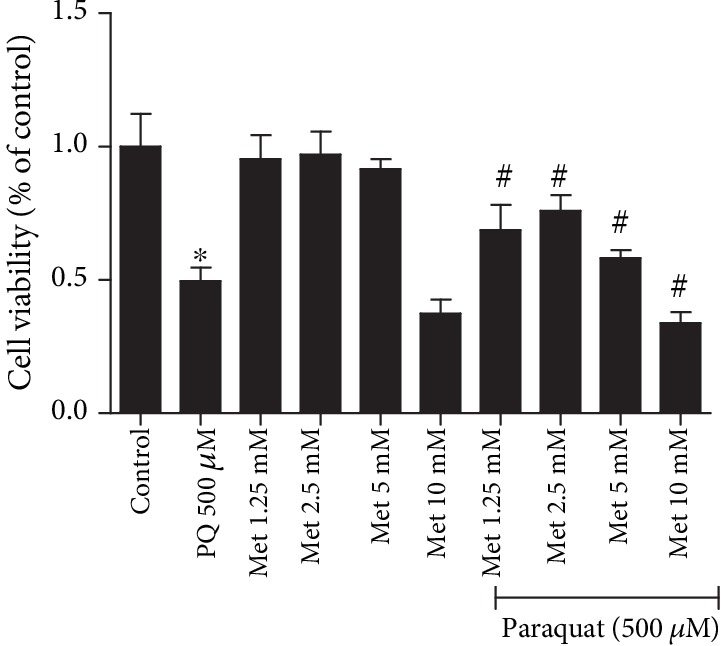
Effect of different concentrations of MET on the viability of rat RLE-6TN cells in the presence of PQ. The cells were pretreated with different concentrations of MET (1.25, 2.5, 5, and 10 mmol/L) for 2 hours and then treated with PQ (500 *μ*mol/L) for 24 hours. Then, 10 *μ*L of CCK-8 solution was added to each well for 2 hours. The data are the average of 6 replicate wells. *F* = 72.344, *P* < 0.001; ^∗^*P* < 0.05 compared with the control group; ^#^*P* < 0.05 compared with the PQ group.

The proliferation rate of RLE-6TN cells after PQ treatment was evaluated by CCK-8 assays. There were significant differences in the effect of different doses of MET on the proliferation rate of RLE-6TN cells after PQ treatment (*P* < 0.05). Cells alone were used as the control group, and the results showed that RLE-6TN cells exposed to MET at 1.25, 2.5, and 5 mmol/L for 24 hours did not have a significant decrease in the cell survival rate. The cell survival rate decreased more obviously in the 10 mmol/L MET group, indicating that MET has no effects on proliferation and no toxicity on cells at concentrations of 1.25, 2.5, and 5 mmol/L but does have a toxic effect on cells at a concentration of 10 mmol/L. After 24 hours of PQ exposure, the cell survival rate was approximately 50%, which was a statistically significant decrease compared with the control group (*P* < 0.001). After pretreatment with 1.25, 2.5, and 5 mmol/L MET for 2 hours and then exposure to PQ for 24 hours, the cell survival rate was higher. The increase in the PQ+MET group compared with the PQ group was statistically significant (*P* < 0.05), and MET activity was optimal at 2.5 mmol/L; thus, this concentration was selected as the intervention dose in subsequent experiments (Figure 6).

### 3.7. Effect of MET on PQ-Induced Changes in Protein Levels in Rat RLE-6TN Cells

**Figure 7:**
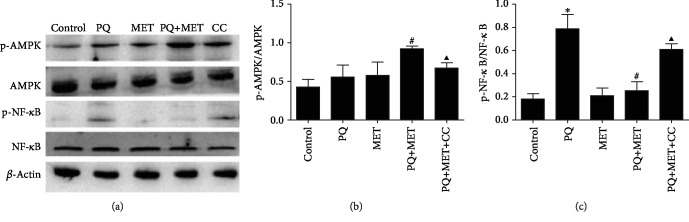
Effect of MET and Com C on the expression of phospho-AMPK and phospho-NF-*κ*Bp65 protein in PQ-treated RLE-6TN cells. The cells were pretreated with MET and Com C for 2 hours and then treated with PQ for 24 hours. Lysis buffer was added to the cells to extract protein for Western blotting analysis. Data are shown as the mean ± SD; (b) *F* = 7.6, *P* < 0.01; (c) *F* = 38.302, *P* < 0.001; ^∗^*P* < 0.05 vs. control group, ^#^*P* < 0.05 vs. PQ group, ^▲^*P* < 0.05 vs. PQ+MET group.

As shown in Figures 7(a)–7(c), the expression of phospho-AMPK protein in RLE-6TN cells was significantly higher in the PQ+MET group than in the PQ group (*P* < 0.01). The expression of phospho-AMPK protein was significantly lower after the addition of the AMPK inhibitor Com C to the PQ+MET group (*P* < 0.05); the expression of phospho-NF-*κ*Bp65 protein in RLE-6TN cells was significantly higher in the PQ group than in the control group (*P* < 0.001) and was decreased after MET intervention (*P* < 0.001). In the PQ+MET+CC group, the expression of phospho-NF-*κ*Bp65 protein was significantly higher than that in the PQ+MET group (*P* < 0.001).

## 4. Discussion

The mechanism by which PQ causes biological poisoning is complicated. It is believed that the mechanism of damage by PQ poisoning mainly involves oxidative stress, mitochondrial damage, immune activation, and inflammatory mediators [4]. Among them, oxidative stress plays an important role in lung injury caused by paraquat [15]. PQ poisoning can induce a large amount of reactive oxygen species (ROS) in the body, generate hydroxyl radicals (-OH), and cause a series of chain reactions such as lipid peroxidation that generate more free radicals, thus leading to the depletion of reducing substances such as NADPH; together, these events trigger lipid peroxidation and damage biofilms, functional proteins, DNA, and structures such as cell membranes and mitochondrial membranes [16]. On the other hand, PQ not only directly causes cellular destruction through lipid peroxidation but also leads to the production of inflammatory cells, such as neutrophils and mononuclear macrophages, that release inflammatory factors, including tumor necrosis factor (TNF-*α*) and interleukins (IL-1*β*, IL-6, and IL-8), which in turn activate inflammatory cascades, triggering systemic inflammatory response syndrome (SIRS) and aggravating tissue damage [17].

In normal cells, nuclear factor-kappa B (NF-*κ*B) binds to inhibitory proteins such as inhibitor of *κ*B and is in a resting state, but ROS can transform NF-*κ*B from a quiescent state to an activated state. The inhibitory protein is rapidly phosphorylated by inducers of NF-*κ*B. Once activated, NF-*κ*B translocates into the nucleus and binds to promoter regions, inducing the transcription of target genes encoding proteins such as inflammatory factors, cytokines, and chemokines, further leading to platelet aggregation, fiber formation, and inflammatory cell accumulation. Therefore, PQ mainly induces cellular damage by ROS production and NF-*κ*B activation to induce nuclear condensation and damage to organelles such as the endoplasmic reticulum.

Studies [18] have also shown that PQ poisoning can induce the release of inflammatory cytokines in human alveolar epithelial A549 cells, with increased expression of TNF-*α*, IL-1*β*, and IL-6. In this study, a rat model of acute lung injury was constructed by PQ exposure. The results confirmed that PQ poisoning can induce inflammatory cell infiltration of the lung tissue in rats (HE staining of pathological sections). Transmission electron microscopy also showed that PQ poisoning can lead to a large amount of inflammatory exudate in the rat alveolar space. The levels of inflammatory factors (TNF-*α*, IL-1*β*, and IL-6) and oxidative end products (MDA) in lung homogenates were also increased, the SOD and GSH-Px activities indicating antioxidant capacity in lung homogenates were decreased, and the above manifestations were alleviated after MET treatment.

As a first-line hypoglycemic agent for the treatment of type 2 diabetes, MET can activate AMPK to reduce oxidative stress [19] and exert anti-inflammatory effects [20]. To investigate the role of MET in PQ-induced ALI, we generated the commonly used PQ-induced rat ALI model [1] to observe the protective antioxidant and anti-inflammatory effects of MET in PQ-induced ALI. Through animal experiments, MET was shown to significantly improve PQ-induced pulmonary edema and reduce oxidative stress and inflammation. Compared with the PQ group, the PQ+MET group showed a significant reduction in the W/D ratio of lung tissue, and the pathological changes in the lung tissue were alleviated, as evidenced by HE staining and transmission electron microscopy. The levels of inflammatory factors (TNF-*α*, IL-1*β*, and IL-6) in lung tissue homogenates were also reduced accordingly. At the same time, after MET treatment, the activities of SOD and GSH-Px in rat lung tissue homogenates increased, and the level of MDA decreased. SOD is an important antioxidant enzyme in the body and a biochemical indicator widely used to reflect oxidative stress [21]. GSH-Px, as an antioxidant enzyme involved in various enzymatic processes within and out of cells, can reduce hydrogen peroxide (H_2_O_2_) and hydroperoxides [22]. MDA is an important metabolite of oxidative stress, and its tissue concentration reflects the severity of oxidative damage in cells [23]. These experimental results indicate that MET has a protective effect against PQ-induced ALI by alleviating oxidative stress and exerting anti-inflammatory activity.

Studies have reported that metformin reduces oxidative stress and has anti-inflammatory effects by activating AMPK-dependent signaling pathways [24, 25]. AMPK is a highly conserved intracellular energy balance sensor that is activated by a decrease in intracellular ATP production, which causes a relative increase in AMP or ADP; AMPK inhibits the anabolic pathway that consumes ATP and promotes the catabolism pathway that produces more ATP to maintain energy and metabolic homeostasis [26]. Recent studies have found that AMPK activity is significantly reduced in LPS-induced ALI. The AMPK activator AICAR can reverse lung inflammatory injury to some extent, and pulmonary infections can be aggravated by the AMPK inhibitor Com C in mice [27]. Similar studies have also found that the activation of AMPK triggers PGC-1*α*-dependent antioxidant responses, thereby reducing mitochondrial ROS production [28], upregulating SOD, and attenuating LPS-induced ALI [29]. These findings are consistent with the results of our study. Our group used Western blotting to show that the expression of phospho-AMPK in the PQ group was lower in the animal model and cellular model. After MET intervention, the expression of phospho-AMPK was significantly increased. After the addition of the AMPK inhibitor Com C, the expression of phospho-AMPK decreased significantly. These data suggested that in the PQ poisoning model, MET can activate the AMPK signaling pathway and can be inhibited by the AMPK inhibitor Com C.

NF-*κ*B is a member of the Rel family of eukaryotic transcription factors, is widely expressed in various mammalian cells, and is considered a key regulator of stress responses, especially inflammatory processes [30]. A large number of studies have shown that inflammatory cells such as neutrophils, macrophages, and lymphocytes aggregate in the lungs in ALI, thereby activating the NF-*κ*B signaling pathway that regulates oxidative stress and inflammatory responses and leading to the release of a variety of inflammatory factors. Inflammatory mediators such as TNF-*α*, IL-1*β*, and IL-6 and adhesion factors ultimately lead to the infinite amplification of inflammatory response cascades [31, 32]. The NF-*κ*B-mediated signaling pathway is an important part of the pathogenesis of ALI [33] and plays an important role in ALI caused by different factors. The results of this study showed that NF-*κ*Bp65 was activated by phosphorylation in the PQ group in animal models and cellular models, resulting in increased protein levels of phospho-NF-*κ*Bp65, significant increases in the oxidation product MDA levels in rat lung homogenates, and obvious decreases in the antioxidant enzyme SOD and GSH-Px activities. Meanwhile, TNF-*α*, IL-1*β*, and IL-6 levels were significantly increased in lung tissue homogenates. After MET treatment, the expression of phospho-NF-*κ*Bp65 protein was significantly decreased, and TNF-*α*, IL-1*β*, IL-6, and MDA levels were significantly decreased in lung homogenates, while SOD and GSH-Px activities were significantly increased. After the addition of the AMPK inhibitor Com C, the expression level of phospho-NF-*κ*Bp65 protein increased once again. These data suggested that AMPK can inhibit the NF-*κ*B signaling pathway after activation, thereby reducing oxidative damage and downregulating inflammatory cytokines.

In summary, our group conducted in vivo and in vitro experiments and showed that MET may upregulate SOD and GSH-Px activities, reduce the oxidation product MDA levels, and downregulate inflammatory factors (TNF-*α*, IL-1*β*, and IL-6) by activating the AMPK/NF-*κ*B signaling pathway, thereby participating in the reduction of oxidative damage and the inhibition of inflammation to protect against PQ-induced ALI. These findings provide new ideas for studying the pathogenesis and clinical treatment of PQ.

## Data Availability

All data generated or analyzed during this study are included in this published article.
